# RETRACTION: Clinical Efficacy and Mechanisms of Microneedling Alone or Combined With Drugs in the Treatment of Androgenetic Alopecia

**DOI:** 10.1111/jocd.70868

**Published:** 2026-04-26

**RETRACTION**: G. Li, J. Geng, J. Liang, J. Gao, L. Ma, Y. Zhang, Y. Huang, B. Zhang, Y. Zhang, J. Shangguan, and S. Liu, “Clinical Efficacy and Mechanisms of Microneedling Alone or Combined With Drugs in the Treatment of Androgenetic Alopecia,” *Journal of Cosmetic Dermatology* 25, no. 2 (2026): e70726, https://doi.org/10.1111/jocd.70726.

The above article, published online on 08 February 2026 in Wiley Online Library (wileyonlinelibrary.com), has been retracted by agreement between the journal Editor‐in‐Chief, Michael H. Gold; and Wiley Periodicals, LLC. The retraction has been agreed due to concerns raised by third parties. Specifically, several cited references were found to be invalid or non‐existent. In response to requests for clarification, the authors repeatedly provided incorrect DOIs and links for the references in question. Accordingly, the article has been retracted, as the concerns were not adequately addressed, leaving several statements in the article unsupported. Furthermore, due to the authors’ continued inability to verify and validate the information provided in their responses to the journal's queries, the editors have concerns regarding possible use of AI‐generated content without appropriate perusal. The authors disagree with the retraction decision.

